# The meaning in life and smart technology of older adults during the Covid-19 pandemic: A cross-cultural qualitative study

**DOI:** 10.1192/j.eurpsy.2022.1272

**Published:** 2022-09-01

**Authors:** S. Von Humboldt, N.M. Mendoza-Ruvalcaba, E.D. Arias-Merino, A. Costa, E. Cabras, G. Low, I. Leal

**Affiliations:** 1 ISPA – Instituto Universitário, Lisbon, Portugal, William James Research Center, Lisbon, Portugal; 2 Universidad de Guadalajara CUTONALA, Health Sciences Division, Guadalajara, Mexico; 3 Universidad de Guadalajara CUCS, Public Health Department, Guadalajara, Mexico; 4 Instituto Universitário, Ispa, Lisbon, Portugal; 5 Universidad Antonio de Nebrija, Departamento De Educación, Madrid, Spain; 6 University of Alberta, Faculty Of Nursing, Edmonton, Canada

**Keywords:** Older Adults, Covid-19 pandemic, Smart technology, Meaning of life

## Abstract

**Introduction:**

The exponential increase of the older segment of the population (1) is coinciding with the growing challenges of a digital society in different socio-cultural contexts (2).

**Objectives:**

This exploratory study aims to analyze older adult perspectives of how smart technology influenced their meaning in life during the Covid-19 Public Health Emergency period, using qualitative research at a cross-national level.

**Methods:**

Three hundred and fifty one community-dwelling older participants aged 65-87 years were included in the study. Participants were Italian, Mexican, Portuguese and Spanish. All the narratives went through a process of content analysis.

**Results:**

Findings of content analysis produced six themes: Meaningful relations, rewarding activities, spirituality, health and safety-related support, self-growth, and physical activity. Smart technology was important in promoting significant relations for Mexican older adults (71.3%), rewarding activities for Portuguese older adults (57.1%), spirituality for Spanish older participants (71.6%), and physical activity for Italian older adults (29.5%).

**Conclusions:**

This study indicated that smart technology during the Health Emergency period was important for the meaning in life of older populations, mostly by facilitating meaningful relations, rewarding activities and spirituality. Future interventions with older adults during pandemic periods should consider the diversity of themes associated with increasing older adult well-being, from a cross-cultural perspective. 1. von Humboldt S & Leal I. The old and the oldest old: Do they have different perspectives on adjustment to aging?. Int J Gerontol; 9:156-160. 2. von Humboldt S et al. Does spirituality really matter? - A study on the potential of spirituality to older adult’s adjustment to aging. *Jpn Psychol Res, 56;*114-125.

**Disclosure:**

No significant relationships.

